# Warm seawater temperature promotes substrate colonization by the blue coral, *Heliopora coerulea*

**DOI:** 10.7717/peerj.7785

**Published:** 2019-09-27

**Authors:** Christine Guzman, Michael Atrigenio, Chuya Shinzato, Porfirio Aliño, Cecilia Conaco

**Affiliations:** 1 Marine Science Institute, College of Science, University of the Philippines Diliman, Quezon City, Philippines; 2 Evolutionary Neurobiology Unit, Okinawa Institute of Science and Technology Graduate University, Onna, Okinawa, Japan; 3 Department of Marine Bioscience, Atmosphere and Ocean Research Institute, The University of Tokyo, Kashiwa-shi, Chiba, Japan

**Keywords:** *Heliopora coerulea*, Blue coral, Transcriptomics, Climate change

## Abstract

**Background:**

*Heliopora coerulea*, the blue coral, is a reef building octocoral that is reported to have a higher optimum temperature for growth compared to most scleractinian corals. This octocoral has been observed to grow over both live and dead scleractinians and to dominate certain reefs in the Indo-Pacific region. The molecular mechanisms underlying the ability of *H. coerulea* to tolerate warmer seawater temperatures and to effectively compete for space on the substrate remain to be elucidated.

**Methods:**

In this study, we subjected *H. coerulea* colonies to various temperatures for up to 3 weeks. The growth and photosynthetic efficiency rates of the coral colonies were measured. We then conducted pairwise comparisons of gene expression among the different coral tissue regions to identify genes and pathways that are expressed under different temperature conditions.

**Results:**

A horizontal growth rate of 1.13 ± 0.25 mm per week was observed for corals subjected to 28 or 31 °C. This growth rate was significantly higher compared to corals exposed at 26 °C. This new growth was characterized by the extension of whitish tissue at the edges of the colony and was enriched for a matrix metallopeptidase, a calcium and integrin binding protein, and other transcripts with unknown function. Tissues at the growth margin and the adjacent calcified encrusting region were enriched for transcripts related to proline and riboflavin metabolism, nitrogen utilization, and organic cation transport. The calcified digitate regions, on the other hand, were enriched for transcripts encoding proteins involved in cell-matrix adhesion, translation, receptor-mediated endocytosis, photosynthesis, and ion transport. Functions related to lipid biosynthesis, extracellular matrix formation, cell migration, and oxidation-reduction processes were enriched at the growth margin in corals subjected for 3 weeks to 28 or 31 °C relative to corals at 26 °C. In the digitate region of the coral, transcripts encoding proteins that protect against oxidative stress, modify cell membrane composition, and mediate intercellular signaling pathways were enriched after just 24 h of exposure to 31 °C compared to corals at 28 °C. The overall downregulation of gene expression observed after 3 weeks of sustained exposure to 31 °C is likely compensated by symbiont metabolism.

**Discussion:**

These findings reveal that the different regions of *H. coerulea* have variable gene expression profiles and responses to temperature variation. Under warmer conditions, the blue coral invests cellular resources toward extracellular matrix formation and cellular migration at the colony margins, which may promote rapid tissue growth and extension. This mechanism enables the coral to colonize adjacent reef substrates and successfully overgrow slower growing scleractinian corals that may already be more vulnerable to warming ocean waters.

## Introduction

The increasing scale and frequency of mass coral bleaching events linked with unusually warm water has greatly contributed to the decline of coral cover across the globe. Since the 1980s, rising sea surface temperatures have resulted in three pan-tropical bleaching events in 1998, 2010 and 2015–2016 (Heron et al., 2016). Recent reports showed that even the most highly protected reefs are not resistant to extreme heat stress (Hughes et al., 2017). Recurrent bleaching leads to less recovery time for corals and, as a consequence, the community structure on some reefs has changed dramatically (Hughes et al., 2017, 2018). If severe bleaching events continue, it is predicted that only 10% of the world’s coral reefs will survive beyond 2050 (Heron et al., 2016). Nevertheless, it has become increasingly evident that coral susceptibility and resilience to bleaching is highly variable (Grottoli et al., 2014; Guest et al., 2012; Marshall & Baird, 2000; Sampayo et al., 2008). Coral genera that are able to withstand or recover from heat stress can repopulate affected reef areas and drive changes in coral reef community structure (Edmunds et al., 2014; Hoegh-Guldberg et al., 2007; Mumby & Van Woesik, 2014).

Corals that are able to tolerate stressors or survive bleaching events are valuable models for revealing the mechanisms underlying differences in resilience (Van Oppen et al., 2015). Analysis of gene expression through transcriptome sequencing provides a means to evaluate the contribution of phenotypic plasticity and local adaptation to the coral environmental response (Kenkel & Matz, 2016). Transcriptome sequencing approaches have revealed high levels of gene expression variation in adult corals from different environments (Barshis et al., 2013; Maor-Landaw et al., 2017), as well as altered expression for many coral genes in response to temperature stress (Bellantuono, Hoegh-Guldberg & Rodriguez-Lanetty, 2011; DeSalvo et al., 2008, 2010a; Kaniewska et al., 2015; Parkinson et al., 2015; Seneca & Palumbi, 2015). Most of these differentially expressed transcripts were derived from the host coral, with only a small proportion originating from the dinoflagellate symbionts (Barshis et al., 2013; Parkinson et al., 2018). Coexpression of genes that function within similar cellular pathways reveal processes that are critical for mounting the coral stress response (Bay & Palumbi, 2017; Rose, Seneca & Palumbi, 2015). Although transcriptome responses vary by species and treatment regime, common biological functions that have been found to be responsive to temperature conditions include protein folding chaperones, removal of damaged macromolecules, redox signaling, apoptosis, calcium homeostasis, and modifications to the actin cytoskeleton and extracellular matrix (DeSalvo et al., 2010a; Kaniewska et al., 2015; Meyer, Aglyamova & Matz, 2011; Parkinson et al., 2015; Seneca & Palumbi, 2015). Resilient corals typically expressed higher levels of thermal tolerance genes, particularly heat shock proteins, antioxidant enzymes, apoptosis regulators, tumor suppressors, innate immune response genes, and cell adhesion molecules (Barshis et al., 2013). It should be noted that most of these studies have been conducted on scleractinian corals, with limited reports for octocorals (Pratlong et al., 2015; Sammarco & Strychar, 2013). Transcriptome sequencing of alcyonacean octocorals, such as *Gorgonia ventalina* and *Corallium rubrum*, revealed expression of immune response genes related to pattern recognition, anti-microbial peptides, and wound repair in response to pathogen exposure (Burge et al., 2013), as well as gene expression signatures of thermal adaptation (Pratlong et al., 2015).

The reef-building octocoral *Heliopora coerulea* is an example of a coral species that survives bleaching events better than most scleractinian corals. Commonly known as the blue coral, *H. coerulea* is thought to be highly resistant to temperature stress and bleaching (Harii et al., 2002; Kayanne et al., 2002; Richards et al., 2018). This coral exhibits considerable morphological plasticity with laminar and digitate forms (Villanueva, 2016; Yasuda et al., 2014), as well as an encrusting form that is observed along colony margins. In contrast, *H. hiberniana*, a newly described *Heliopora* species from north Western Australia, has a distinctive slender branching growth form with a white skeleton (Richards et al., 2018).

*Heliopora coerulea* is found in the Indo-Western Pacific region between 25°N and 25°S (Zann & Bolton, 1985). Specifically, *H. coerulea* thrives in waters with a mean annual minimum temperature above 22 °C, which is considerably higher than the 18 °C marginal isotherm for many corals (Zann & Bolton, 1985). Recently, the northernmost populations of *H. coerulea* have been discovered in Tsukazaki, Japan where the lowest temperature is around 18 °C (Nakabayashi et al., 2017). *H. coerulea* can dominate large reef areas although, in most cases, its colonies are patchily distributed due to its short larval duration (Atrigenio, Aliño & Conaco, 2017; Harii et al., 2002). It has also been observed that adult *H. coerulea* can inhibit the settlement of other scleractinian larvae in its vicinity (Atrigenio, Aliño & Conaco, 2017). In the Bolinao-Anda Reef Complex in northwestern Philippines facing the South China Sea, *H. coerulea* coral cover has increased from just 1% in the 1990s to about 50% after 20 years (Atrigenio, 1995; Vergara, 2009), during which time two mass bleaching events were reported (Arceo et al., 2001; Shaish et al., 2010). The increasing prevalence of *H. coerulea* coincides with rising sea surface temperature in the South China Sea region, which was estimated at 0.50 ± 0.26 °C per decade from 1993–2003 (Fang et al., 2006) and 0.31 °C per decade from 2003–2017 (Yu et al., 2019) based on high-resolution satellite data. The ability of *H. coerulea* to compete for space on the reef may be attributed to various factors, chief among which could be resistance to environmental stressors affecting the area, which include temperature variability (Penaflor et al., 2009), reduced salinity (Cardenas et al., 2010), and eutrophication (Ferrera et al., 2016). However, little is known about how these factors influence the growth of *H. coerulea*. Furthermore, no studies have yet been conducted to examine gene expression dynamics in this coral under different temperature conditions.

To understand the molecular mechanisms governing the response of *H. coerulea* to varied temperatures, we observed the growth rate of the coral at temperatures spanning the typical range experienced at the study site. We then analyzed changes in the patterns of gene expression both in the coral host and its symbionts. The findings of this study reveal the underlying processes that control the rapid growth of *H. coerulea* over the reef.

## Materials and Methods

### In situ coral overgrowth measurements

Overgrowth measurements were done on coral colonies found within a 10 × 10 m area at three to five m depth in Lucero, Bolinao, Pangasinan, Philippines (N 16°2441, E 119°5420) from January to December 2016. A total of 10 *H. coerulea* colonies naturally growing over massive *Porites* sp. were randomly selected and tagged with numbered pieces of aluminum. Horizontal growth of the *H. coerulea* colony was measured by taking the distance from the growing edge to a permanent reference line marked by two concrete nails hammered onto the reef floor near the colony. Growth measurements were taken every 2 weeks for 1 year. Two loggers (HOBO) were deployed near the site for continuous monitoring of temperature.

### Coral collection and temperature-controlled experiments

Coral colonies (~7.5 cm in diameter) were collected by SCUBA diving at three to five m depth in Lucero, Bolinao, Pangasinan, Philippines (N 16°2441, E 119°5420) in 2015 with permission from the Philippines Department of Agriculture Bureau of Fisheries and Aquatic Resources (DA-BFAR GP-0097-15). Independently growing colonies of *H. coerulea* (digitate form), as well as colonies of *H. coerulea* growing over massive *Porites* sp. (encrusting form), were collected using hammer and chisel. Corals were glued to tiles (7.5 cm^2^) underwater and were left for a 2-week healing period at the collection site. Corals were then transported to the Bolinao Marine Laboratory and acclimated in flow-through seawater tanks at 28 °C for another week. Corals were randomly placed into independently-aerated glass aquaria containing flow-through sand-filtered seawater. Temperature in experimental tanks was gradually lowered or raised (0.25 °C h^−1^) to the desired setting using using chillers (Hailea 1HP) or submersible aquarium heaters (Eheim Jaeger 300W). Corals were maintained at three temperatures that were selected to span the typical temperature range recorded in Bolinao: 26 ± 1 °C represents the average temperature during the coldest months of the year, 28 ± 1 °C represents the yearly average temperature, and 31 ± 1 °C represents the average temperature during the warmest months of the year. Three replicate tanks were used for each temperature regime. Each tank contained six corals consisting of three digitate colonies and three encrusting colonies (a total of 54 colonies for each experimental run). Temperature-controlled experiments were performed with two outdoor runs conducted from January to March 2015 (trials 1 and 2). Due to difficulties in maintaining temperature stability in the outdoor experimental tanks, a third experimental run was conducted indoors from June to July 2015 (trial 3). Samples for sequencing of digitate tissues were collected from corals in the outdoor experiments under shaded natural sunlight of ~1,000 lux. The margin and encrusting tissues were collected from corals from the indoor experiment conducted under ~500 lux illumination on a 12:12 light-dark cycle provided by 20W LED daylight lamps.

Water temperature in the tanks was monitored using temperature probes (Vernier LabQuest) and underwater temperature and light loggers (HOBO) set to record every 30 min. Horizontal growth at the edges of *H. coerulea* growing over a flat portion of *Porites* was measured using a plastic caliper with a set reference point. Photosynthetic efficiency was measured using a pulse amplitude-modulated fluorometer (Diving-PAM; Walz Inc., Effeltrich, Germany). Coral fragments were collected after 24 h or 3 weeks of exposure. White margin tissues were carefully separated from adjacent calcified encrusting tissues using surgical scissors. Coral fragments were immediately flash-frozen in liquid nitrogen for transport and subsequent RNA extraction.

### RNA extraction and sequencing

Total RNA was extracted using Trizol (Invitrogen, Carlsbad, CA, USA) following the manufacturer’s protocol. For digitate colonies, coral fragments (~2.5 cm length) from each coral colony were manually homogenized using a mortar and pestle. The new growth and calcified encrusting tissues of *H. coerulea* colonies growing over *Porites* were dissected and treated as separate tissue samples. Contaminating genomic DNA in the RNA extracts was removed using the Turbo DNA-free kit (Life Technologies, Carlsbad, CA, USA) followed by ethanol precipitation. Nucleic acid concentrations were quantified using the Qubit 3.0 fluorometer (Life Technologies, Carlsbad, CA, USA). RNA integrity was assessed by electrophoresis on native agarose gels with denaturing loading dye and using an Agilent Bioanalyzer 2100. Duplicate or triplicate samples were selected for RNA sequencing. Total RNA samples were sent to the Beijing Genomic Institute, Hong Kong, for mRNA enrichment and preparation of barcoded libraries using the Illumina TruSeq RNA Sample Prep Kit V2 protocol. A total of 20 libraries were sequenced on the Illumina HiSeq 2000 platform with 100-bp paired-end reads (Table S1).

### Gene abundance estimation and differential expression analyses

Raw sequence reads were filtered to remove adapter sequences and low-quality reads. Trimmomatic 0.32 (Bolger, Lohse & Usadel, 2014) was used to trim the first 10 bases of the reads. Reads were scanned using a four-base sliding window, deleting bases with average quality below 20. Bases with quality scores below 30 at leading and trailing ends were also trimmed. Only reads that passed the quality filters and that were longer than 30 bases were retained for further analysis. Gene abundance estimation for each sequence library was performed by mapping paired-end reads back to a previously assembled metatranscriptome of *H. coerulea* (Guzman et al., 2018) using the RNASeq by expectation maximization with Bowtie2 alignment method included in the Trinity package suite (Grabherr et al., 2011; Langmead & Salzberg, 2012; Li & Dewey, 2011).

Analysis of differentially expressed transcripts was conducted on edgeR (Robinson, McCarthy & Smyth, 2010) using counts obtained from the abundance estimation. Only transcripts with greater than one count per million in at least two samples were included in the analysis. Genes that were up or downregulated with a log_2_ fold change > |2| and a *p*-value < 0.05 (Benjamini–Hochberg adjusted) were considered differentially expressed. Samples used for edgeR comparisons are listed in Table S1. Differentially expressed transcripts are listed in Data S1.

### GO enrichment analysis and PFAM domain annotation

The *H. coerulea* transcriptome assembly was previously annotated by alignment to the RefSeq database at an *e*-value cutoff of 1 × 10^−5^. The top blastx hit for each gene was used as input into Blast2GO (Conesa et al., 2005) to retrieve gene ontology (GO) terms. Enrichment analysis for differentially expressed genes was performed using the topGO package in R (Alexa & Rahnenfuhrer, 2018). Only GO terms with a *p*-value < 0.05 were considered significantly enriched. Protein domains were identified by mapping predicted peptides against the Pfam 28.0 database using HMMER v3.1b1.

## Results

### Physiological response of *H. coerulea* to various temperature regimes

**Figure 1:**
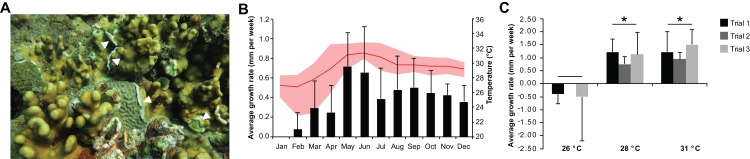
*Heliopora coerulea* grows rapidly over the reef. (A) *H. coerulea* growing over other scleractinian corals. The new growth is visible as white tissues at the margins or edges of the colony (white arrowheads). (B) In situ monitoring of *H. coerulea* growth over the course of a year in relation to recorded seawater temperature at the reef site. Growth is shown in average millimeters per week (mm/week). Error bars represent standard deviation of measurements from replicate colonies. Average monthly temperature is indicated by the red line and the shaded area represents the minimum and maximum recorded temperatures. (C) Colony margins extend at a faster rate at 28 and 31 °C compared to 26 °C. Growth is shown in average millimeters per week (mm/week) for three experimental runs. Error bars represent standard deviation of measurements from replicate colonies. Asterisks indicate significant differences relative to the 26 °C treatment (*p*-value < 0.005, ANOVA and Tukey’s test).

*Heliopora coerulea* exhibited a rapid horizontal growth rate at warmer temperature (Fig. 1A; Fig. S1). An average horizontal growth rate of 0.41 ± 0.18 mm per week was recorded from in situ monitoring of *H. coerulea* colonies over the course of a year (Fig. 1B; Data S2). During this observation period, the average seawater temperature at the reef site was 29.36 ± 1.49 °C, ranging from a minimum of 22.87 °C to a maximum of 32.87 °C. Maximum growth rate reached 0.65–0.72 mm per week in the months of May–June 2016, coinciding with a seawater temperature average of 31.25 ± 0.17 °C (Fig. 1B). On the other hand, slowest growth rates were observed from January to April when temperatures averaged 27.73 ± 1.12 °C at the site. From April to July, maximum temperatures recorded at the site reached >32 °C, which resulted in bleaching of several coral genera. Quantitation of bleaching in June 2016 revealed that, although *H. coerulea* constituted about 32% of the coral cover in the area, only 5% of its colonies showed visible signs of bleaching (Fig. S2). In contrast, 14–71% of the colonies of six other scleractinian species comprising 4–20% of the coral cover in the study area exhibited bleaching.

In the controlled temperature experiments, *H. coerulea* colonies growing over *Porites* exhibited an average growth rate of 1.13 ± 0.25 mm per week when exposed to temperatures from 28 to 31 °C, which was significantly faster than growth at the colder temperature of 26 °C (ANOVA *p*-value = 0.0007; Fig. 1C; Data S2). Growth rates observed in the controlled experiments were higher than what was measured in the field likely due to the stability of temperature conditions in the aquaria. The effect of temperature on horizontal growth rates was similar for experiments conducted at different times of the year in either outdoor or indoor aquaria. *H. coerulea* colonies did not show any obvious signs of bleaching stress, such as whitening of tissue and necrosis, after 3 weeks of exposure to the various temperature treatments. In addition, maximum photochemical yields (Fv/Fm), which ranged from 0.68 to 0.73, did not change significantly during the 3 weeks of exposure to different temperatures (Fig. S3).

### Global transcriptome profiles

**Figure 2:**
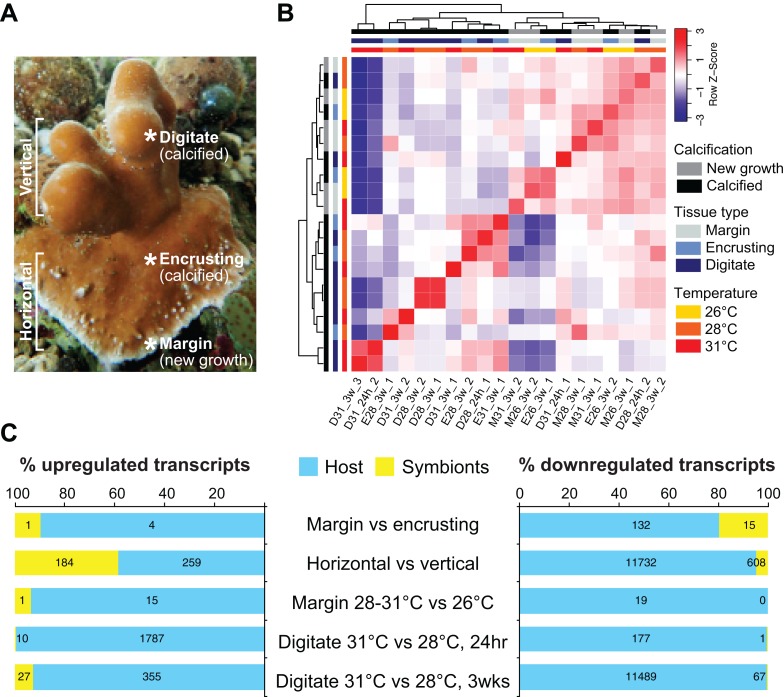
Transcriptome profiling of *H. coerulea*. (A) Different regions of an *H. coerulea* colony from which tissues were collected for transcriptome sequencing. (B) Correlation of overall expression profiles of samples representing different tissues and treatments. (C) The proportion of up or downregulated genes originating from either coral host (blue) or symbionts (yellow) in each of the indicated comparisons. Numbers of differentially expressed transcripts under each classification are shown on the bars.

We sequenced and analyzed the transcriptomes of different tissues (Fig. 2A) taken from coral colonies that had been incubated under various temperature conditions. Comparison of global transcriptome profiles revealed greater similarity with respect to tissue type rather than to temperature treatment. The calcified tissues of *H. coerulea*, including both encrusting and digitate regions, clustered together in the correlation matrix (Fig. 2B). The margin tissues also formed a separate cluster, although this was interspersed with some samples of calcified encrusting tissues from the 26 °C treatments. The calcified digitate tissues from corals that had been subjected to different temperatures for only 24 h showed variable clustering with the other samples. Variability between biological replicates may reflect inter-colony differences in gene expression responses. It is important to note that this correlation matrix was generated using the full metatranscriptome, which includes transcripts originating from the coral host and its symbionts.

### Differential gene expression across coral regions

**Figure 3:**
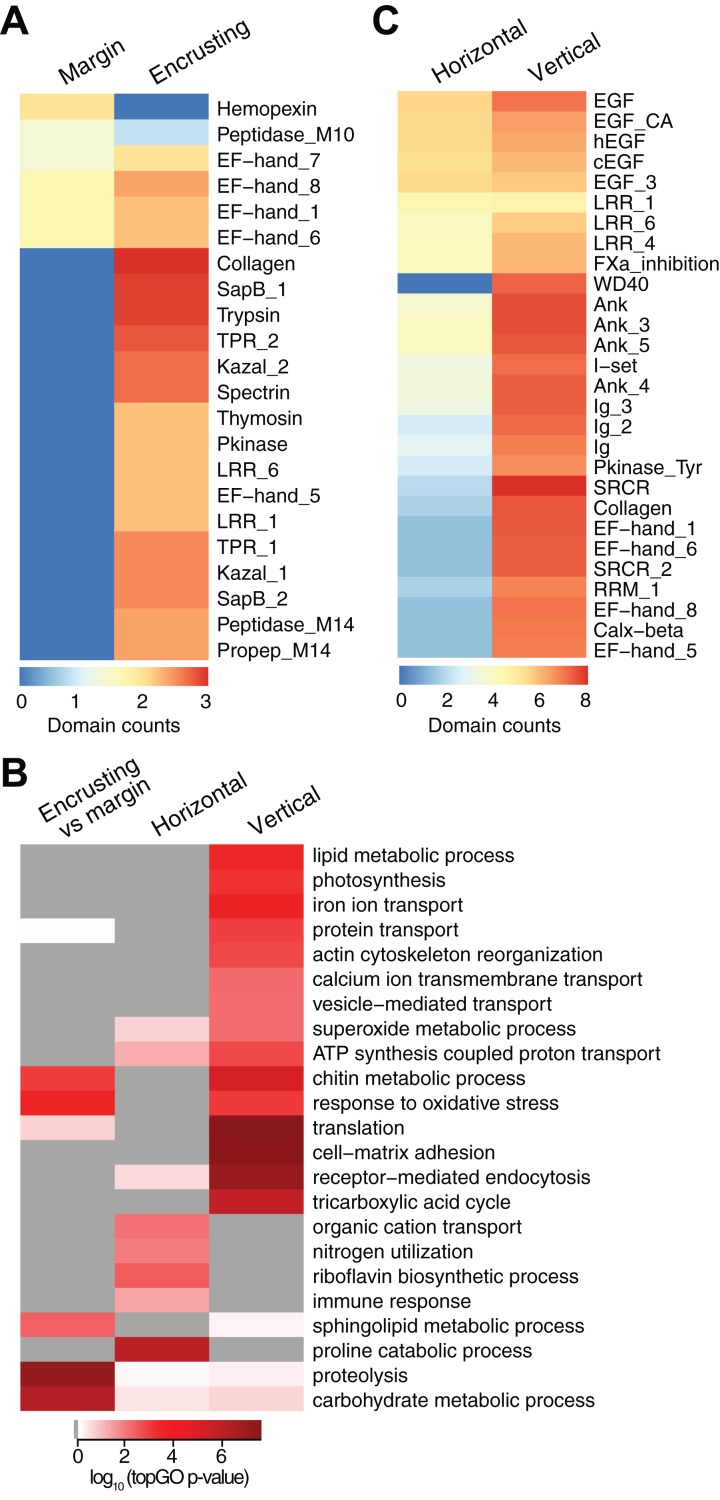
Counts of PFAM domains and enriched gene ontology terms in predicted peptides encoded by differentially expressed transcripts in different tissue regions. PFAM domain counts in margin vs encrusting tissues (A) and in horizontal vs vertical growth regions (C). Heatmap colors represent abundance of each PFAM domain (red, high; blue, low). (B) Enriched gene ontology terms in different tissue regions. Heatmap colors represent log_10_ of the topGO enrichment *p*-value (red, high; white, low; gray, not enriched).

New growth at the margins of *H. coerulea* colonies consisted of whitish tissue that is more flexible to the touch compared to adjacent calcified encrusting tissues. Only five transcripts were more highly expressed in the margin tissues relative to the calcified encrusting region, of which one is a matrix metallopeptidase with hemopexin domains, another is a calcium and integrin binding (CIB) protein with multiple EF-hand domains, and three are unknown sequences (Fig. 3A; Data S3). A total of 147 transcripts were significantly upregulated in the calcified encrusting region relative to the margin and this set is enriched for collagen, trypsin, SapB, TPR, Kazal, EF hand, peptidase M14, protein kinase, and LRR domains (Fig. 3A; Data S3). Enriched functions in the calcified encrusting region include proteolysis, carbohydrate metabolic process, response to oxidative stress, chitin metabolic process, and sphingolipid metabolic process (Fig. 3B; Data S4).

Compared to the vertical (digitate) growth region, 443 transcripts were more highly expressed in the horizontal growth region, that includes both margin and calcified encrusting areas, with 41.53% of the transcripts originating from symbionts and 58.47% from the host (Fig. 2C; Table S2). These transcripts are enriched for epidermal growth factor (EGF), leucine-rich repeat (LRR), and ankyrin domains (Fig. 3C; Data S3). The encoded genes are enriched for functions related to biosynthesis of proline and riboflavin, nitrogen utilization, and organic cation transport (Fig. 3B; Data S4). On the other hand, 12,783 transcripts were found to be more highly expressed in the vertical (digitate) region of the coral compared to the horizontal region, with only 4.93% originating from symbionts and 95.07% from the host. Common domains found in these transcripts include SRCR, ankyrin repeats, EF hand, immunoglobulin, collagen, and EGF (Fig. 3C; Data S3). These domains are associated with functions related to cell-matrix adhesion, translation, receptor-mediated endocytosis, chitin metabolism, vesicle-mediated transport, photosynthesis, carbohydrate and lipid metabolic processes, actin cytoskeleton reorganization, and calcium and iron ion transport (Fig. 3B; Data S4). Interestingly, transcripts encoding homologs of biosynthetic enzymes for biliverdin IXα, the blue pigment found in the *H. coerulea* skeleton (Hongo, Yasuda & Nagai, 2017), were detected in all tissues examined (Fig. S4). Most of these transcripts were expressed at higher relative abundance in the calcified encrusting and vertical (digitate) regions, whereas biliverdin reductase, which reduces biliverdin to bilirubin, was detected only in the vertical region.

### Effect of temperature on differential gene expression at the margin

**Figure 4:**
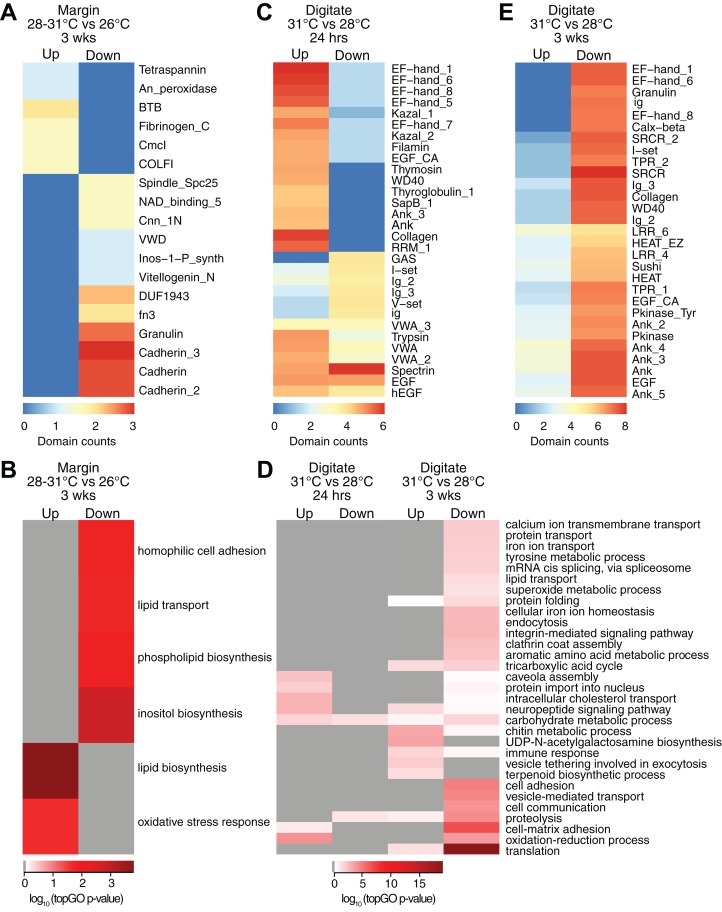
Counts of PFAM domains and enriched gene ontology terms in predicted peptides encoded by differentially expressed transcripts in tissue regions of corals subjected to different temperatures. PFAM domain counts in margin tissues subjected to 28–31 vs 26 °C for 3 weeks (A) digitate tissues at 31 vs 28 °C for 24 h (C) and digitate tissues at 31 vs 28 °C for 3 weeks (E). Heatmap colors represent abundance of each PFAM domain (red, high; blue, low). Enriched gene ontology terms in margin tissues subjected to 28−31 vs 26 °C for 3 weeks (B) and digitate tissues at 31 vs 28 °C for 24 h or 3 weeks (D). Heatmap colors represent log_10_ of the topGO enrichment *p*-value (red, high; white, low; gray, not enriched).

Controlled temperature experiments revealed higher horizontal growth rates for *H. coerulea* colonies kept at 28 and 31 °C compared to controls at 26 °C. To identify transcripts that correspond to this enhanced growth, we compared the transcriptome of margin tissues obtained from colonies subjected to 28 and 31 °C to margin tissues from colonies kept at 26 °C. Exposure to temperatures of 28 or 31 °C for up to 3 weeks resulted in upregulation of 16 transcripts and downregulation of 19 transcripts at the margin compared to corals subjected to 26 °C (Fig. 2C; Table S2). Upregulated transcripts encoded proteins containing CmcI methyltransferase, tetraspanin, fibrillar collagen, peroxidasin, and BTB domains (Fig. 4A; Data S3), with enrichment for functions related to lipid biosynthesis, extracellular matrix formation, and response to oxidative stress (Fig. 4B; Data S4). On the other hand, downregulated transcripts contain cadherin, granulin, and fibronectin domains (Fig. 4A; Data S3) and are predicted to be involved in cell adhesion, lipid synthesis and transport, and inositol metabolism (Fig. 4B; Data S4).

### Effect of temperature on differential gene expression in the digitate region

Transcriptome profiles in the digitate region of the coral were examined to elucidate the effect of temperature levels typically experienced during the warmest months of the year on *H. coerulea* gene expression. Digitate tissues from coral colonies subjected to 31 °C (summer average) were compared to tissues from colonies kept at 28 °C (yearly average). After 24 h of exposure to 31 °C, 1,797 transcripts were upregulated relative to corals maintained at 28 °C (Fig. 2C; Table S2). Overrepresented PFAM domains include EF hand, collagen, ankyrin, and trypsin (Fig. 4C; Data S3). Enriched functions include oxidation-reduction, neuropeptide and intracellular signaling, protein import, and carbohydrate metabolic process (Fig. 4D; Data S4). Only 178 transcripts were downregulated at 24 h, with overrepresentation of spectrin, EGF, and immunoglobulin domains (Fig. 4C; Data S3).

After 3 weeks of sustained exposure to 31 °C, 382 transcripts were upregulated in the digitate region, 7.07% of which originated from the symbionts (Fig. 2C; Table S2). Common domains represented in this set of transcripts are ankyrin and LRR repeats (Fig. 4E; Data S3). Enriched functions are related to the chitin metabolic process, immune response, neuropeptide signaling, tricarboxylic acid cycle, and exocytosis (Fig. 4D; Data S4). In contrast, 11,556 transcripts were downregulated under these conditions, with only 0.58% originating from symbionts (Fig. 2C; Table S2). This set of transcripts represents a wide array of cellular functions, including translation, cell and cell-matrix adhesion, oxidation-reduction, vesicle-mediated transport, iron ion homeostasis, and carbohydrate metabolic process (Fig. 4D; Data S4). The set of downregulated transcripts were enriched for EF hand, protein kinase, ankyrin, collagen, SRCR, WD40, sushi, and EGF domains (Fig. 4E; Data S3).

## Discussion

*Heliopora coerulea* can tolerate prolonged exposure of up to 3 weeks to conditions approximating the mean summer temperature at the site. Blue coral colonies did not show any obvious signs of bleaching stress in the form of tissue whitening and necrosis or tissue sloughing throughout the entire duration of the experiments. Results of the controlled temperature experiments were supported by field observations that showed less prevalence of bleaching for *H. coerulea* colonies compared to scleractinian corals in the same area. Similar observations on the bleaching resistance of *H. coerulea* have been reported by other groups (Harii et al., 2002, 2014; Kayanne et al., 2002; Richards et al., 2018; Shaish et al., 2010).

*Heliopora coerulea* colonies were observed to grow faster over substrate when subjected to warm temperatures (28 or 31 °C) than when exposed to cold (26 °C), both in situ and in aquaria with controlled seawater temperature. Corals subjected to 26 °C revealed low or negative growth at the margins, suggesting loss or shrinkage of living tissue. This suggests that the distribution of *H. coerulea* may be limited by its apparent susceptibility to cooler temperatures, although more studies are needed to investigate the cold tolerance limits of this species. The average in situ horizontal growth rate of *H. coerulea* was 0.41 ± 0.18 mm per week (approximately 21 mm per year) and could reach 0.65–0.72 mm per week during the warmest months of the year. These rates of growth are greater than what has been reported for massive *Porites* (about 0.20 per week or approximately 10 mm per year) (Lough & Barnes, 2000). In the eastern Pacific, growth rates ranging from 13.9 to 19.3 mm per year were reported for the encrusting growth form of *Porites lobata*, although this rapid growth was shown to correlate only with longer light period and higher salinity but not with differences in temperature (Guzmán & Cortés, 1989). The correlation between *H. coerulea* growth and temperature suggests that, under future ocean conditions where temperatures may increase by 1.2–3.2 °C (IPCC, 2014), *H. coerulea* will have the advantage over slower-growing scleractinians in colonizing available reef substratum.

Coral growth and mineralization is biologically controlled and involves cycles of extension and skeletal thickening (Cuif & Dauphin, 2005). Extension is the secretion of a mineralizing matrix consisting of a mixture of proteins, polysaccharides, and glycoproteins, while skeletal thickening is the crystallization of mineral material onto the organic framework. The spatial organization of the organic framework upon which calcification occurs is determined by cell-cell and cell-substrate adhesion mediated by the extracellular matrix, which contains collagen and cadherins (Helman et al., 2008; Mass et al., 2014). Cytoskeletal components, such as actin, control cell shape and are important for vesicular transport and cellular movement (Svitkina, 2018). Thus, differential activation of growth and mineralization processes in corals can be inferred through comparative transcriptome profiling across different tissue regions or across different treatment conditions.

Transcriptome analysis revealed that the vertical digitate and horizontal extension regions of the coral exhibit very different expression profiles. However, these tissues show an enrichment of symbiont-related functions, such as photosynthesis, carbohydrate metabolism, nitrogen utilization, superoxide metabolic process, and ATP synthesis, suggesting that symbiont metabolism is active throughout the coral. In the vertical region, enrichment of calcium ion transport and iron ion sequestration functions may be related to calcification of the blue-pigmented *H. coerulea* skeleton. Some transcripts encoding enzymes for the synthesis of the blue pigment, biliverdin IXα (Hongo, Yasuda & Nagai, 2017), were relatively more abundant in the vertical region of the coral. Enrichment of chitin metabolism in the vertical region indicates its importance in formation of the coral skeleton. Chitin, along with other sulfur-containing proteins, has been shown by Raman spectroscopy to form part of the organic matrix that controls aragonite crystal size, shape, and orientation in the fibers of the *H. coerulea* skeleton (Zhang et al., 2011).

Tissues at the *H. coerulea* growth margin were enriched for transcripts that may enhance cell migration. For example, the matrix metallopeptidase with hemopexin domains has been reported to promote cell migration through a non-proteolytic mechanism in epithelial cells (Dufour et al., 2008). The CIB protein, on the other hand, is known to positively regulate cell migration and focal adhesion complex formation (Naik & Naik, 2011). Furthermore, upon exposure to warmer temperatures, transcripts encoding proteins with roles in extracellular matrix formation were upregulated, including peroxidasin and fibrillar collagen. Peroxidasin is an oxidative stress response gene that is often reported as differentially expressed in many coral heat stress studies (Barshis et al., 2013; Louis et al., 2017; Voolstra et al., 2011). In myofibroblasts, peroxidasin is secreted into the extracellular space where it helps form the extracellular matrix as a means of wound repair and tissue fibrosis (Peterfi et al., 2009). Peroxidasin has also been reported to be strongly upregulated during symbiont colonization of coral tissue (Yuyama et al., 2018). Fibrillar collagens, on the other hand, provide mechanical strength and stability to tissues. The unique arrangement of fibrillar collagens in octocorals is an important hydroskeleton structure to support soft coral tissues (Orgel et al., 2017). This suggests that warmer temperature may promote the production of proteins required for the extracellular matrix and cell migration at colony margins. The organic matrix laid down by migrating cells at the colony margin serves to promote nucleation of aragonite crystals that eventually build up its massive skeleton. The ability to shift from one form to another in order to compete for substrate has been previously observed in the scleractinian coral, *Montipora aequituberculata*, which can overgrow the sponge, *Terpios hoshinota*, by shifting from foliose to encrusting morphology (Elliott et al., 2015). Interestingly, genome sequencing of *Montipora capitata*, a coral that also exhibits an encrusting morphology at its base, revealed enrichment for functions related to proteinaceous extracellular matrix, collagen trimer, and cell-matrix adhesion in the set of genes under diversifying selection (Shumaker et al., 2019). Proliferation of the encrusting or plating growth form of some corals may eventually lead to reduction of reef rugosity and complexity (Magel et al., 2019).

Upregulation of transcripts encoding proteins involved in the extracellular matrix, cytoskeleton, and cell migration or morphogenesis has been reported in other scleractinian corals subjected to elevated temperature conditions, although these studies did not look specifically at transcripts expressed at colony growth margins. Upregulation of extracellular matrix genes were observed in *Acropora palmata* subjected to 2 °C above ambient for 24–48 h (DeSalvo et al., 2010b), as well as in *A. hyacinthus* subjected to 4 °C above ambient for 5 or 20 h (Seneca & Palumbi, 2015). However, in contrast to the upregulation of collagens at the growth margin of *H. coerulea* subjected to 28 to 31 °C for 3 weeks, collagen transcripts were downregulated in heat-stressed *A. hyacinthus* (Seneca & Palumbi, 2015). In addition, enrichment of functions related to translation, adhaerens junction, cytoskeleton, and morphogenesis were observed in *Stylophora pistillata* exposed to 2 °C above ambient for 1 week (Maor-Landaw et al., 2017). On the other hand, decreased cytoskeletal and cell adhesion functions were reported in *Montastrea faveolata* subjected to 3 °C above ambient for up to 9 days (DeSalvo et al., 2008). Similarly, *Galaxea fascicularis* subjected to 7 °C above ambient for 18 h showed downregulation of transcripts involved in the regulation of cell migration and cell morphogenesis (Hou et al., 2018). In these latter two studies, the downregulation of cytoskeleton, cell adhesion, and cell migration functions may be linked to tissue damage and visible coral bleaching. It should be noted, however, that the differences in gene expression response reported in these studies may be due to the different treatment conditions that were used. Further studies that apply similar conditions, approximating either natural temperature maxima or bleaching thresholds, to investigate the differential response of diverse coral species are warranted.

The calcified digitate tissues of *H. coerulea* exhibited a diverse gene expression profile, with enrichment for genes that function in translation, cell-matrix adhesion, oxidation-reduction processes, photosynthesis, carbohydrate metabolic process, and calcium and iron ion transport. This indicates that calcified tissues of the coral are invested in energy generation and biomineralization, with photosynthetic activity of the symbionts playing a central role. It is likely that the enrichment of oxidative response genes in calcified coral tissues is a mechanism to counteract the negative effects of reactive oxygen species generated by the photosynthetic activity of the symbionts (Baird et al., 2009).

Short-term exposure (24 h) to 31 °C resulted in upregulation of transcripts encoding peptides involved in oxidation-reduction, carbohydrate metabolic processes, cholesterol transport, and neuropeptide signaling. These changes suggest that short term fluctuations in temperature may trigger signaling cascades that induce expression of transcripts encoding proteins that protect against oxidative stress, modify cell membrane composition, and mediate intercellular signaling pathways that potentially modify coral behavior. Upregulation of protective oxidation-reduction enzymes and signaling pathways is consistent with observations in other corals subjected to heat stress (Barshis et al., 2013; DeSalvo et al., 2008; Seneca & Palumbi, 2015).

Three weeks of exposure to 31 °C resulted in a global decline in gene expression, as demonstrated by the downregulation of thousands of transcripts. This effect may be attributed to the reallocation of energy towards activation of other cellular pathways, the elevated cost of basal metabolism, and inhibition of pathways for energy generation as the organism nears its thermal tolerance limits (Kaniewska et al., 2015; Sokolova, 2013). Nevertheless, we observed upregulation of some transcripts involved in immune response, protein catabolism and vesicle exocytosis, which may be linked to cellular repair mechanisms that are induced by protein denaturation under warmer conditions. Sustained exposure to 31 °C also resulted in upregulation of metabolic functions that originate from the symbionts, such as tricarboxylic acid cycle. This suggests that symbiont functions remained intact under the treatment conditions, which is supported by the lack of significant change in photosynthetic efficiency. In fact, only a few symbiont sequences showed significant transcriptional response to 31 °C in *H. coerulea* and a greater proportion (7.07%) were found in the upregulated set as compared to the downregulated set of transcripts (0.58%). This is in agreement with other reports indicating that stress triggers a greater shift in gene expression in the coral host rather than in the symbionts *in hospite* due to a host buffering effect (Barshis et al., 2014; Leggat et al., 2011).

Taken together, our results revealed that *H. coerulea* is able to withstand temperatures of up to 31 °C. Although sustained exposure to this condition resulted in a general decline in gene expression, coral symbionts appeared to remain functional. This suggests that they continue to provide energy to the coral host and thus support the rapid tissue extension that is observed at colony margins. Horizontal growth of the blue coral colony was correlated with enhanced expression of cell matrix, cytoskeleton, and cell migration transcripts at the growth margins. By prioritizing tissue growth and colony margin extension, the blue coral can continue to colonize substrate even under conditions that may be stressful to many scleractinian corals. Whether this enhanced growth is sustained at temperatures above 31 °C warrants further studies.

## Conclusions

Continued warming of the oceans has resulted in declining coral growth and calcification rates (Cantin et al., 2010; Cooper et al., 2008). Under present and future ocean scenarios, only corals with a high thermal resistance, rapid growth, and low mortality are likely to persist (Edmunds et al., 2014). The ability of *H. coerulea* to rapidly grow over substrates under warmer seawater conditions allow it to outcompete slower-growing reef organisms for benthic space. This may have contributed to the present-day dominance of the blue coral at the study site in the Bolinao-Anda Reef Complex in northwestern Philippines. Other factors, such as a reduction in coral recruitment or an increase in the mortality of other corals, may have also contributed to changes in coral community structure at the study site.

Although the persistence of *H. coerulea* may compensate for some ecosystem functions that are lost due to the decline of scleractinians (Richards et al., 2018), the long-term impact of *H. coerulea* dominance on other reef-associated marine organisms remains to be determined. Competition with *H. coerulea* can negatively affect the growth, fecundity, and survival of other coral species (Romano, 1990). *H. coerulea* may also inhibit scleractinian coral recruitment through allelopathy or other mechanisms (Atrigenio, Aliño & Conaco, 2017). Moreover, *H. coerulea* may have a different calcification rate that could limit its ability to contribute to reef growth (Perry et al., 2015). Further studies are needed to investigate the tolerance limits of *H. coerulea* to temperature, as well as to other local stressors, such as salinity shifts and eutrophication. It would also be important to examine the interactions of the blue coral with other reef biota to understand how its dominance may ultimately affect reef biodiversity.

## Supplemental Information

10.7717/peerj.7785/supp-1 Supplemental Information 1 Supplementary Figures and Tables.

10.7717/peerj.7785/supp-2 Supplemental Information 2 Differentially expressed transcripts from pairwise comparisons of tissues or temperature treatments. Annotations are based on best hits to UniProt and RefSeq databases are shown, as well as associated gene ontology terms and PFAM domains. Transcript abundance in each library is represented as counts per million reads (CPM).

10.7717/peerj.7785/supp-3 Supplemental Information 3 Horizontal growth of *H. coerulea* colonies on the reef (in situ) and in controlled laboratory experiments (ex situ) reported as millimeters per week (mm/week). Temperature readings from a logger (HOBO) deployed at the reef site are also shown.

10.7717/peerj.7785/supp-4 Supplemental Information 4 PFAM domain counts from the set of predicted peptides encoded by differentially expressed transcripts from comparisons across tissues or temperatures.

10.7717/peerj.7785/supp-5 Supplemental Information 5 Gene ontology enrichment for up or downregulated transcripts in comparisons between tissues or temperatures. Only GO terms with a topGO enrichment *p*-value < 0.05 were considered significant.

## Additional Information and Declarations

### Field Study Permissions

The following information was supplied relating to field study approvals (i.e., approving body and any reference numbers): Coral colonies were collected by SCUBA diving in Lucero, Bolinao, Pangasinan, Philippines with permission from the Philippines Department of Agriculture Bureau of Fisheries and Aquatic Resources (DA-BFAR GP-0097-15).

### DNA Deposition

The following information was supplied regarding the deposition of DNA sequences: The data used in this paper is available in the NCBI Short Read Archive: BioProject PRJNA397991.

### Data Availability

The following information was supplied regarding data availability: The raw data is available in the Supplemental Files.
