# Supplementary material for: Warm seawater temperature promotes substrate colonization by the blue coral, Heliopora coerulea
Source: PeerJ. 2019 Sep 27;7:e7785. doi: 10.7717/peerj.7785 (PMC6768060; doi:10.7717/peerj.7785)
Supplement: Supplemental Information 1 [file peerj-07-7785-s001.docx]

**Supplementary Materials**

**Warm seawater temperature promotes substrate colonization by the blue coral, *Heliopora coerulea***

**Christine Guzman^1,2^, Michael Atrigenio^1^, Chuya Shinzato^3^, Porfirio Aliño^1^, and Cecilia Conaco^1*^**

^1^Marine Science Institute, College of Science, University of the Philippines, Diliman, Quezon City 1101, Philippines

^2^Evolutionary Neurobiology Unit, Okinawa Institute of Science and Technology Graduate University, Onna, Okinawa 904-0495, Japan

^3^Department of Marine Bioscience, Atmosphere and Ocean Research Institute, The University of Tokyo, Kashiwa-shi, Chiba 277-8564, Japan

*Corresponding author: Cecilia Conaco, cconaco@msi.upd.edu.ph


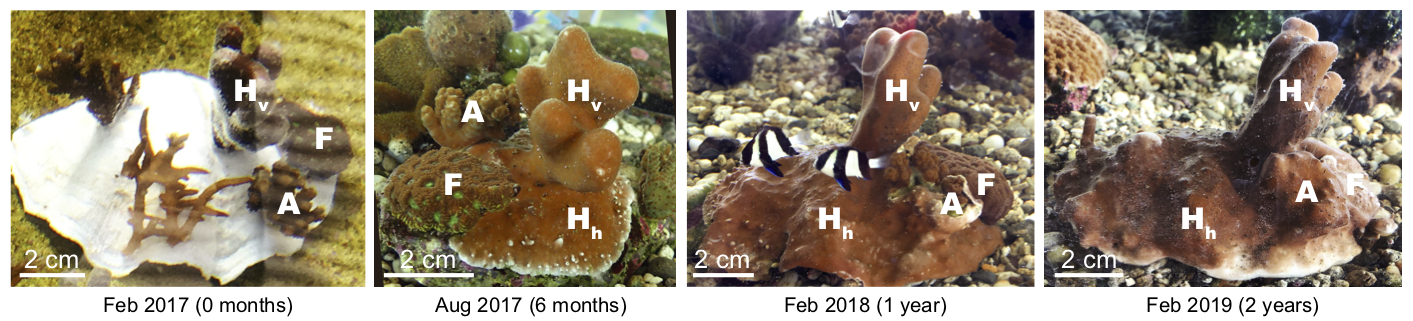


**Figure S1.** Growth of a *H. coerulea* fragment attached to an artificial substrate. An *H. coerulea* fragment (Hv) attached to a giant clam shell exhibited rapid horizontal growth (Hh) that was already apparent within 6 months of transplantation. Within one year of transplantation, the coral had completely covered all available bare substrate. After two years, the coral had also covered adjacent colonies of *Acropora tenuis* (A) and *Favites colemani* (F). The other coral fragments attached to the shell had died or were dislodged before the 6 month image was taken. Scale bars represent 2 cm.

**Figure S2.** Coral community composition and bleaching occurrence in Lucero, Bolinao, Pangasinan, in June 2016. Coral community composition at genus level is indicated by the colored pie chart while bleaching prevalence for colonies from each genus is represented by the smaller pie charts (gray, bleached; black, not bleached). Data was obtained from two 50 m transects at 3-5 m depth.

**Figure S3.** Average photosynthetic efficiency (Fv/Fm) of *H. coerulea* colonies (digitate) upon exposure to different temperatures for the indicated durations. No significant change in photosynthetic efficiency was detected under the experimental treatments. Error bars represent standard deviation of replicate colonies.

**Figure S4.** Expression of enzymes of the heme biosynthesis pathway in different coral tissue regions. Homologous sequences in the *H. coerulea* transcriptome were identified by blast alignment against reference sequences for each enzyme. The average expression of transcripts in replicate libraries derived from margin, encrusting, or vertical (digitate) tissues was computed and the average values were summed across all transcripts belonging to each gene family. Heatmaps represent log_2_ of transcripts per million (TPM) +1 (red, high; dark blue, low).

**Table S1.** Transcriptome libraries used for differential expression comparisons. Coral region, tissue type, temperature treatment, and time of sampling are indicated.

| **Library** | **Region** | **Tissue type** | **Temperature** | **Time** |
| --- | --- | --- | --- | --- |
| Calcified encrusting vs margin | | | | |
| IM26_3w_1 | horizontal | margin | 26°C | 3 weeks |
| IM26_3w_2 | horizontal | margin | 26°C | 3 weeks |
| IM28_3w_1 | horizontal | margin | 28°C | 3 weeks |
| IM28_3w_2 | horizontal | margin | 28°C | 3 weeks |
| IM31_3w_1 | horizontal | margin | 31°C | 3 weeks |
| IM31_3w_2 | horizontal | margin | 31°C | 3 weeks |
| IO26_3w_1 | horizontal | calcified encrusting | 26°C | 3 weeks |
| IO26_3w_2 | horizontal | calcified encrusting | 26°C | 3 weeks |
| IO28_3w_1 | horizontal | calcified encrusting | 28°C | 3 weeks |
| IO28_3w_2 | horizontal | calcified encrusting | 28°C | 3 weeks |
| IO31_3w_1 | horizontal | calcified encrusting | 31°C | 3 weeks |
| Vertical vs horizontal region | | | | |
| IM26_3w_1 | horizontal | margin | 26°C | 3 weeks |
| IM26_3w_2 | horizontal | margin | 26°C | 3 weeks |
| IM28_3w_1 | horizontal | margin | 28°C | 3 weeks |
| IM28_3w_2 | horizontal | margin | 28°C | 3 weeks |
| IM31_3w_1 | horizontal | margin | 31°C | 3 weeks |
| IM31_3w_2 | horizontal | margin | 31°C | 3 weeks |
| IO26_3w_1 | horizontal | calcified encrusting | 26°C | 3 weeks |
| IO26_3w_2 | horizontal | calcified encrusting | 26°C | 3 weeks |
| IO28_3w_1 | horizontal | calcified encrusting | 28°C | 3 weeks |
| IO28_3w_2 | horizontal | calcified encrusting | 28°C | 3 weeks |
| IO31_3w_1 | horizontal | calcified encrusting | 31°C | 3 weeks |
| IN31_3w_1 | vertical | digitate | 31°C | 3 weeks |
| IN31_3w_2 | vertical | digitate | 31°C | 3 weeks |
| IN28_3w_1 | vertical | digitate | 28°C | 3 weeks |
| IN28_3w_2 | vertical | digitate | 28°C | 3 weeks |
| Margin 28-31°C (warm) vs 26°C (cool), 3weeks | | | | |
| IM26_3w_1 | horizontal | margin | 26°C (cool) | 3 weeks |
| IM26_3w_2 | horizontal | margin | 26°C (cool) | 3 weeks |
| IM28_3w_1 | horizontal | margin | 28°C (warm) | 3 weeks |
| IM28_3w_2 | horizontal | margin | 28°C (warm) | 3 weeks |
| IM31_3w_1 | horizontal | margin | 31°C (warm) | 3 weeks |
| IM31_3w_2 | horizontal | margin | 31°C (warm) | 3 weeks |
| Digitate 31°C vs 28°C, 24hr | | | | |
| IN28_24h_1 | vertical | digitate | 28°C | 24hr |
| IN28_24h_2 | vertical | digitate | 28°C | 24hr |
| IN31_24h_1 | vertical | digitate | 31°C | 24hr |
| IN31_24h_2 | vertical | digitate | 31°C | 24hr |
| Digitate 31°C vs 28°C, 3weeks | | | | |
| IN28_3w_1 | vertical | digitate | 28°C | 3 weeks |
| IN28_3w_2 | vertical | digitate | 28°C | 3 weeks |
| IN31_3w_1 | vertical | digitate | 31°C | 3 weeks |
| IN31_3w_2 | vertical | digitate | 31°C | 3 weeks |
| IN31_3w_3 | vertical | digitate | 31°C | 3 weeks |

**Table S2.** Differentially expressed transcripts across tissues or temperature treatments. The numbers of up or downregulated transcripts classified as originating from either the host coral or the symbionts are shown for each comparison.

| Comparison | Upregulated | | Downregulated | |
| --- | --- | --- | --- | --- |
|  | Coral | Symbiont | Coral | Symbiont |
| Margin vs calcified encrusting | 4 | 1 | 132 | 15 |
| Horizontal vs vertical region | 259 | 184 | 11732 | 608 |
| Margin 28-31°C vs 26°C, 3weeks | 15 | 1 | 19 | 0 |
| Digitate 31°C vs 28°C, 24hr | 1787 | 10 | 177 | 1 |
| Digitate 31°C vs 28°C, 3weeks | 355 | 27 | 11489 | 67 |
